# Risk, sanctions and norm change: the formation and decay of social distancing norms

**DOI:** 10.1098/rstb.2023.0035

**Published:** 2024-03-11

**Authors:** Eva Vriens, Giulia Andrighetto, Luca Tummolini

**Affiliations:** ^1^ Institute of Cognitive Sciences and Technologies, Italian National Research Council, Via S. Martino della Battaglia 44, 00185 Rome, Italy; ^2^ Institute for Futures Studies, Holländargatan 13, 11136 Stockholm, Sweden; ^3^ Institute for Analytical Sociology, Linköping University, 60174 Norrköping, Sweden

**Keywords:** norm change, social expectations, meta norms, risk, COVID-19, social distancing

## Abstract

Global challenges like the climate crisis and pandemic outbreaks require collective responses where people quickly adapt to changing circumstances. Social norms are potential solutions, but only if they themselves are flexible enough. The COVID-19 pandemic provided a unique opportunity to study norm formation and decay in real-world contexts. We tracked empirical and normative expectations about social distancing and empirical and normative expectations of sanctioning from June 2021 to February 2022 to explore how norms and meta norms evolved as COVID-19 risk decreased and increased. We found that norms and meta norms partially coevolve with risk dynamics, although they recover with some delay. This implies that norms should be enforced as soon as risk increases. We therefore tested how sanctioning intentions vary for different hypothetical norms and find them to increase with a clear meta norm of sanctioning, yet decrease with a clear social norm of distance. In conclusion, social norms evolve spontaneously with changing risk, but might not be adaptive enough when the lack of meta norms of sanctioning introduce tolerance for norm violations. Moreover, norm nudges can potentially have negative externalities if strengthening the social norm increases tolerance for norm violations. These results put some limits to social norms as solutions to guide behaviour under risk.

This article is part of the theme issue ‘Social norm change: drivers and consequences’.

## 1. Introduction

Social norms are often praised as solutions to overcome social dilemmas where individuals face incentives to free-ride on the efforts of others [1]. They provide behavioural rules that are understood by members of the same group and guide or constrain behaviour in that group without having to rely on the force of laws [2]. The presumption is that norms make behaviour stable, because (i) they make it easier to coordinate and predict what others will do [3] and (ii) they come with a fear of social sanctions in case of non-compliance [4]. For social norms to be effective, however, there needs to be a shared agreement around the norm. That is, predictability arises from the (shared) expectations that a sufficiently large subset of others complies with the norm (empirical expectations) and believes one should comply (normative expectations) [5]. The fear of sanctions, while sometimes considered an implicit part of normative expectations, can depend on the existence of a meta norm of norm enforcement [6–8] that people comply with based on the expectation that a sufficiently large subset of others may sanction norm violations and believe that one should (i.e. empirical and normative expectations of sanctioning).

In standard (stable) social dilemma settings, such social expectations are developed and solidified over time, strengthening the norm and stabilizing behaviour. Social norms in these settings are internalized: people do not think of their behaviour as an act of norm compliance [9]. However, many of the most pressing societal, environmental and health challenges we face nowadays require collective action under uncertain and changing circumstances. This requires new, dynamic, or context-specific social norms to develop. Climate change, for instance, brings severe risks that, while their exact magnitude is uncertain, may vary in severity over time. Think, for instance, of seasonal climate risks like wildfires and droughts that make collective efforts to restrict water use more urgent during the warm summer months [10]. Likewise, the COVID-19 pandemic required a collective response in terms of improved hygiene, social distancing and vaccination, but the severity of the risk—and thus the importance of behavioural change—varied over time, with several waves of high infection and mortality rates [11].

Given the large scale and (potentially) fast development of such collective risks, top-down institutional arrangements are often insufficient, unenforceable or too costly [12]. The nationwide lockdowns and school closures that many countries imposed during the first wave of the pandemic, for instance, were effective [13], but also bore extreme economic [14] and social costs [15]. Intuitively, this gives an important role to (informal) social norms to complement or replace more general institutional measures [1,16], for they seem to be more adaptive. Yet despite a large literature showing the potential of social norms to motivate, for instance, sustainable food choices, energy conservation or social distancing [17,18], less is known about how social norms themselves are affected by the changing context, potentially compromising their effectiveness in solving dilemmas [19]. In these settings, it is plausible that social norms are less internalized and that compliance instead remains a more deliberate act that is conditional on one’s expectations about the behaviour and beliefs of other people [20].

The COVID-19 pandemic, for instance, required a rapid shift from deep-rooted, internalized and stable norms (such as shaking hands when meeting new people) to new norms that prevent physical contact and promote distance instead. These norms, which serve to decrease the COVID-19 infection and mortality risk, were needed only as long as infection and mortality rates were high. Yet what happens to these norms when there are several recurrences of high risk? Ideally, the norms are conditional on risk levels and there is a shared agreement on reactivating the norm every time the risk levels require it. In practice, however, the changing context may introduce additional uncertainty not just around the need for a specific behaviour (should people continue to keep distance when the COVID-19 risk decreases?), but also around the expected behaviour and normative beliefs of others: will others continue to keep their distance? Do they think that you should? Will they still sanction violations?

Hence, in a changing context, the two core traits that make social norms successful drivers of behaviour—their predictability and the fear of sanctions—may be undermined. It is not evident that dynamic norms, established in settings characterized by change and uncertainty, invoke compliance to the same extent as stable social norms [21,22]. To leverage social norms for social dilemmas under risk and uncertainty we need to better understand how norms evolve. Do they weaken when risk decreases? And, more importantly, can they recover spontaneously when risk recurs? The COVID-19 pandemic has provided us with a unique opportunity to study processes of norm formation and norm decay in real-world contexts within relatively short time frames. Specifically, tracking norms throughout the pandemic yields insight into the effectiveness of norms in changing contexts—particularly when the contextual change may result in changes in the norm itself (i.e. cycles of norm formation and decay).

We collected data about social expectations of social distancing and sanctioning of people living in Rome (Italy) in two periods: a period of COVID-19 risk decrease (June–August 2021) and a period of risk increase (October 2021–February 2022). Our goal was to study whether norms and meta norms of social distancing covary with risk. During the last two waves, we also presented respondents with hypothetical situations to test whether different norms invoke changes in sanctioning behaviour. Using the strategy method, we manipulated the social norm of distance and meta norm of sanctioning and asked how they would behave in each scenario. We used the scenario of keeping distance in line at the supermarket. While some of the social norms on preventative measures were legally enforced (e.g. closing restaurants and stores when risk is too high), the social norm of keeping distance from others is purely socially enforced. Governments did declare a minimum distance that should be kept from others in public places (in Italy this was at least 1 m), but had no means to legally enforce this rule. Instead, a shared agreement about the importance of maintaining distance from one another to minimize the risk of infection had to be created bottom-up.

## 2. Theory

### (a) From risk to norms

The COVID-19 pandemic has been characterized by several waves of high infection and mortality rates (i.e. high risk). Throughout the pandemic, the Italian government stipulated that people should maintain at least 1 m distance from one another in public places. However, it is by no means guaranteed that people indeed expected others to (believe one should) comply with this rule throughout this whole period. We tracked perceived social norms and meta norms in Rome (Italy) in the aftermath of the second wave (as COVID-19 risk was decreasing) and from the start towards the peak of the third wave (as COVID-19 risk increased again). We hypothesize that while officially the same rule was in place this entire period, people changed their expectations about the distance others actually keep (empirical expectations of distance), the distance others should keep (normative expectations of distance), the distance at which other people start to protest and intervene (empirical expectations of sanctioning), and the distance at which other people believe one should intervene (normative expectations of sanctioning). Specifically, we expect that people change their expectations as a result of changes in the COVID-19 risk. Since there is no coherent theory about the dynamics between (changing or recurring) risk, social norms and meta norms in short time frames yet, we formulate two contrasting hypotheses based on the evolutionary theory of tightness–looseness [23] and the compliance–violation asymmetry bias [24,25].

The tightness–looseness theory of culture [23] poses that from an evolutionary perspective, societies that have experienced more ecological and social threats (e.g. frequent disease, warfare and environmental catastrophes) throughout their history have developed tight cultures, regulated by strong social norms and low tolerance of deviant behaviour, to maintain order and survive chaos and crisis. In low-threat societies, instead loose cultures arose, where norms are weak to non-existing and social sanctions rare. Multiple cross-sectional surveys [23,26,27] and ethnographic studies [28] have shown that countries do in fact vary in the tightness of social norms and that this variation correlates with the prevalence of threats in these countries’ histories [26]. During the COVID-19 pandemic, nations with high levels of cultural tightness and which faced more threats in the past were also characterized by a lower number of cases and deaths compare to nations with higher levels of cultural looseness [29].

Importantly, however, this theory explains cross-country differences in the strength of social norms from an evolutionary perspective. Little is known about processes of tightening and loosening of social norms *within* countries over time. During the COVID-19 pandemic, the level of threat posed by the pandemic changed constantly due to exogenous seasonal influences [30], mutations of the COVID-19 coronavirus, the adherence to non-pharmaceutical and behavioural interventions, and, once vaccines became available, the rates of vaccination uptake [31]. Therefore, the question is whether social norms were able to adapt to these changes in real-time by loosening up when risk decreased but quickly regaining a level of high norm conformity and low tolerance for deviance when a new wave started.

In the abstract context of a Collective Risk Social Dilemma experiment, it has been shown that social norms indeed get stronger in contexts of high threats, but norms also weakened when the risk of threats decreased [32]. Whether norms recover again when facing another recurrence of high threat was not explored. Following the logic of tightening and loosening, it is possible that changes in risk create an oscillatory dynamic between risk and social norms: when risk decreases, it becomes less urgent to comply with the social norms relevant to cope with the risk and violations become widespread; but once the risk increases again, norms tighten up quickly.

On the other hand, the compliance–violation asymmetry bias [24,25] suggests that people respond differently to observations of norm compliance and norm violations. Specifically, people react more strongly to (few) instances of norm violations than to (many) instances of norm compliance. As a result, to strengthen expectations about social norms many acts of compliance need to be observed, but only a few violations are sufficient to break them down. If we follow this asymmetry, one could expect that as risk decreases and norm compliance is less needed, norm violations quickly become widespread and people adjust their social expectations accordingly. A similar dynamic may also affect meta norms since observing more violations and fewer responses to norm violations may also weaken the social expectations supporting sanctions. If these expectations for sanctioning are weak or not existent, this might be an obstacle for the retrieval of social norms if risk were to increase again. If social and meta norms get ‘too loose’ [33], it will be difficult for social and meta norms to recover rapidly when the risk rises again and loose norms might be ineffective to promote cooperation.

Both theories predict that social and meta norms decrease when risk decreases. We thus hypothesize that social norms of distance and meta norms of punishment decrease as the COVID-19 risk decreases (H1). For the evolution of social norms when the COVID-19 risk returns, we formulate two alternative hypotheses. Following the tightness–looseness theory of culture, we expect that norms are adaptive (H2a): social expectations distance and of sanctioning positively associate with risk such that they decrease when the risk decreases and increase when risk increases again. Following the compliance–violation asymmetry bias we expect instead that norms are erosive (H2b): social expectations of distance and sanctioning decrease when risk decreases, but do not increase when risk increases again (i.e. no association between risk and social expectations).

### (b) From norms to behaviour

Government-wide guidelines mandating the minimal distance to be maintained from others have been issued since the beginning of the COVID-19 pandemic. However the ubiquity and small-scale nature of everyday interactions made it difficult, and often impossible, to legally enforce them. As a consequence, social distancing had to rely almost exclusively on informal social sanctions. Therefore we also use norm nudges to test how the presence or the absence of social norms of distance and meta norms of sanctioning may affect sanctioning intentions. Norm nudges are interventions aimed to change behaviour through the manipulation of social expectations [34–36]. That is, norm nudges tell subjects what the norm in place is to see if that changes their behaviour or behavioural intentions. Norm nudges rely on the assumption that presenting information to subjects that a strong norm is in place increases the compliance with the norm [3,5,37]. However, we do not know whether providing information on the social norm or on the meta norm influences sanctioning behaviour in the same way as it does general social norm compliance.

On the one hand, if a strong social norm is in place this may also signal the importance of sanctioning violations (if any), so we should expect a positive effect. This has been found, for instance, in the context of dishonesty [38], where strengthening the anti-lying norm increased norm enforcement. On the other hand, if a strong norm is in place it may be assumed that one’s response to the few expected violations is less necessary or that one can rely on others to do so. First, this may be because sanctioning is typically costly and because people dislike those who sanction [39]. A strong norm may dissuade individuals from wasting resources and risking the cycle of retaliation that it is often initiated by punishing in the first place [40]. Second, while personally costly, sanctioning often also benefits others [41], and as a consequence it is subject to the problem of diffusion of responsibility when others are expected to provide it [42,43]. Strengthening the norm may in fact lead people to believe that others already sanction, thus reducing the personal willingness to enforce it. In other words, there is a risk that norm-based interventions may backfire (see also [44] of this theme issue)

Therefore, we test how norm nudges influence norm enforcement behaviour by comparing both the effect of norm nudges changing the social norm and norm nudges changing the meta norm. While the mechanisms behind norm nudges for norm enforcement may well be different from those for norm compliance, we start from the standard norm nudges logic and hypothesize that positive nudges increase sanctioning intentions in case of norm violations such that: (H3a) strengthening the meta norm of sanctions increases sanctioning, while weakening the meta norm of sanctions decreases sanctioning; (H3b) strengthening the social norm of distance increases sanctioning, while weakening the social norm of distance decreases sanctioning.

## 3. Methods

### (a) Data

Data were collected among respondents from the Qualtrics panel, filtering on respondents that live in Rome (Italy). The first round of data collection took place between 6 June and 11 August 2021, a period of COVID-19 risk decrease. In this period, we surveyed every two weeks a new cross-sectional sample of respondents. The second round of data collection lasted from 13 October 2021 to 3 February 2022, a period of COVID-19 risk increase. In this period, we surveyed a new cross-sectional sample of respondents every four weeks (table 1). This resulted in a pooled cross-sectional dataset of 1977 respondents in total. In all waves, the sample was stratified by gender (50% men, 50% women) and age (evenly distributed across the three categories, less than 30, 30–50 and more than 50). To minimize differences between respondents in the extent to which they are informed about the latest COVID-19 developments, the first page of the survey informed respondents about COVID-19 statistics on infection, mortality and vaccination rates in Lazio (the Italian region of which Rome is the capital) for the two weeks preceding the data collection (see electronic supplementary material, section S1).

**Table 1.** Period of data collection and number of respondents per wave.

wave	date start	date end	*N*
1	9 June 2021	10 June 2021	110
2	23 June 2021	24 June 2021	110
3	7 July 2021	10 July 2021	220
4	21 July 2021	23 July 2021	220
5	4 Aug 2021	11 Aug 2021	217
6	13 Oct 2021	18 Oct 2021	220
7	17 Nov 2021	19 Nov 2021	220
8	15 Dec 2021	16 Dec 2021	220
9	12 Jan 2022	14 Jan 2022	220
10	2 Feb 2022	3 Feb 2022	220
total			1977

We then asked them about social distancing and other COVID-related behaviour and social norms. Since compliance is driven by what people experience on a daily basis in their direct environment, we wanted to measure the expectations about the local social norm in place and therefore restricted our survey to people living in Rome. Using a local reference population makes the norm more salient and simplifies the formulation of social expectations [45,46]. Following Bicchieri *et al.* [47], we incentivized the answers by eliciting the social expectations for Rome specifically and comparing the reported social expectations to the aggregate behaviour and beliefs of all participants to the survey. Each wave, the respondent whose estimates were closest to the aggregate reported answers was rewarded a bonus of €25.

### (b) Measures

**Figure 1.**
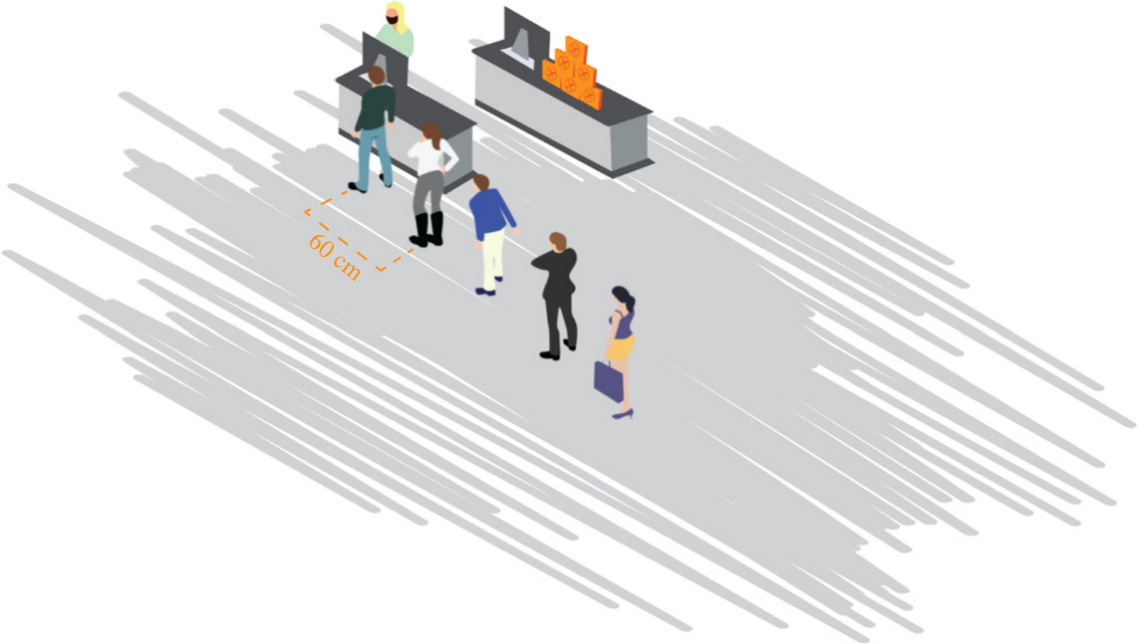
Visualization of people standing in line in the supermarket used to elicit social expectations. By moving a slider, participants could move the distance between the people in line between 40 cm and 160 cm. (Online version in colour.)

As an indicator of social distancing, we took the distance in line at the checkout of an indoor supermarket. Similar to Fazio *et al.* [48], we used a visual representation of people standing in line (figure 1). Using a slider, respondents could change the distance between the people to any distance between 40 cm and 160 cm. The visualization facilitates the interpretation of physical distance and should result in more reliable answers [48]. We used the same continuous scale of physical distance to measure people’s social expectations about the average distance other people living in Rome keep when waiting in line (empirical expectations: EE), the distance other people think one should keep (normative expectations: NE), the distance at which they think other people would intervene and ask the violator to take a step back (empirical expectations of sanctioning), and the distance at which they believe others think people should intervene (normative expectations of sanctioning). Measuring all expectations on the same continuous scale allows one to contrast dynamics both across expectations and within expectations over time. See electronic supplementary material (section S2) for the precise question wording.

As an indicator for COVID-19 risk at the time of the survey we took the mortality in the two weeks preceding the data collection. We opted for mortality rather than infection, because compared to mortality the infection rate increased tremendously in the second period of data collection (October–February), when the Omicron mutation became dominant. Hence, infection rates were no longer a good indicator of COVID-19 risk. We log-transformed the variable to correct for its exponential increase that would have resulted in a skewed distribution otherwise.

To test the hypotheses that manipulating the social norm and the meta norm change sanctioning behaviour, we asked people to imagine that one person in line keeps less than 1 m distance and another person asks that person to please take a step back. To 220 participants in wave 9 we asked ‘Would you respond in the same way if you knew that the majority of people that lives in Rome [*would be ready to intervene/would not be ready to intervene*] if they found themselves in a similar situation’ (manipulation of descriptive norm of sanctions) and ‘Would you respond in the same way if you knew that the majority of people that lives in Rome [*finds it appropriate to intervene/does not find it appropriate to intervene*] if they found themselves in a similar situation’ (manipulation of injunctive norm of sanctions). To 220 participants of wave 10, we asked ‘Would you respond in the same way if you knew that the majority of people that lives in Rome keeps a distance of [*less than 1 m/more than 1 m*] in line at the supermarket’ (manipulation of descriptive norm of distance) and ‘Would you respond in the same way if you knew that the majority of people that lives in Rome believes one should keep a distance of [*less than 1 m/more than 1 m*] in line at the supermarket’ (manipulation of injunctive norm of distance).

We calculate whether people report different intentions to sanction if the meta norm of sanctioning or the social norm of distance were different by taking the difference between their baseline sanctioning behaviour (*does intervene/does not intervene* if someone kept a distance of less than 1 m) and the reported willingness to sanction in the hypothetical scenario. The resulting variable is 0 if the respondent did not change their behaviour compared to the baseline, 1 if they start to sanction but did not before, and −1 if they stop sanctioning in the hypothetical scenario. If, in response to any of these hypothetical scenarios, people changed their sanctioning intentions compared to their self-reported sanctioning behaviour, we asked them to explain why they did. While by no means exhaustive, these open answers may give some indications of possible mechanisms for the link between social norms, meta norms and behaviour.

### (c) Analytical strategy

For each of the four dependent variables (EE/NE of distance and EE/NE of sanctioning), we estimate three multilevel ordinary least squares (OLS) regression models (with respondents nested in waves) to test whether there is a positive association between COVID-19 risk and the dependent variable (DV). To test Hypothesis 1, Model M1 tests for each of the four DVs whether COVID-19 risk significantly explains the content of social expectations in waves 1–5 (the period of COVID-19 risk decrease). If the relationship between COVID-19 risk and social expectations were perfect, expectations would respond to risk increase the same way as they do to risk decrease. That is, the coefficient would be the same for this partial model with half of the waves and for the full model with all waves. Before we estimate the same regression model on the full data, we therefore extrapolate on the findings of Model M1 to predict how high each of the social expectations would be in waves 6–10 given (i) the mortality in waves 6–10 and (ii) the relation between mortality and the DV in waves 1–5. That is, we calculate $E(DV_{W})={\mathrm{intercept}}_{DV,M1}+b{\left(\mathrm{Log}(\mathrm{New}\;\mathrm{deaths}\;t-1)\right)}_{DV,M1}\times \mathrm{Log}{\left(\mathrm{Newdeaths}\;t-1\right)}_{W}$. This gives us an indication of what the social expectations would look like if they were to follow risk equally under risk decrease and risk increase.

Subsequently, we explore whether social expectations indeed covary with COVID-19 risk (Hypothesis 2a) or instead erode (Hypothesis 2b) using full data (waves 1–10). Since we observe qualitatively that there is a slightly increasing trend in waves 6–10 that lags behind the expected trend based on the extrapolation of waves 1–5, we test this in two models. Model 2a has the same specifications as Model 1 and thus presents a pure test of Hypothesis 2a. Model 2b recognizes that the increase in social expectations might follow the increase in COVID-19 risk with some delay and therefore includes a dichotomous control variable ‘Period 2’ that takes the value 1 in waves 6–10 (and 0 otherwise). This control variable serves to correct the intercept at the start of Period 2 (the period of risk increase).

We then proceed to test whether sanctioning intentions increase after strengthening the meta norm of sanctioning (Hypothesis 3a) or the social norm of distance (Hypothesis 3b) using as DVs the reported change in sanctioning intentions in response to any of the hypothetical scenarios. Four OLS regression models estimate whether sanctioning increases for four dependent variables ([*descriptive/injunctive*] norm of [*distance/sanctioning*]) and for each of the two scenarios ([*norm is present: majority does/norm is not present: majority does not*]). All models include a correction of the standard errors to control for the clustering of scenarios within respondents. Since the DVs have three values (−1, 0, 1), we estimate, for each model, the average marginal effects of change in sanctioning intentions in response to these two scenarios to obtain the overall probability of behavioural change. Subsequently, we explore what drives the overall results by including an interaction between the scenario and the baseline sanctioning behaviour to calculate the probability of behavioural change for people who already indicated to sanction and people who do not.

All analyses control for gender (male, female, other), age, educational degree (6-item low–high), political preferences (7-item left–right), perceived health (6-item poor–good), previous COVID-19 infection (yes–no), risk preferences (11-item risk averse–risk seeking), generalized trust (11-item low–high), trust in politicians (5-item low–high), trust in doctors (5-item low–high) and trust in scientists (5-item low–high). All continuous variables are mean-centred to obtain a more meaningful value of the intercepts. The tables present the main results with respect to our hypotheses; detailed results about the effects of the covariates are presented in the electronic supplementary material. The data and scripts to reproduce the results are publicly available on OSF (https://osf.io/6fh4v).

## 4. Results

### (a) From risk to norms

**Figure 2.**
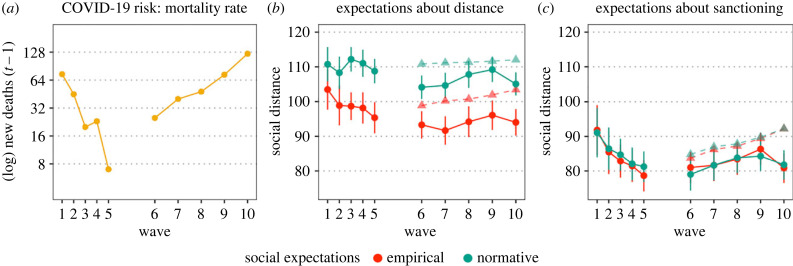
Trends in COVID-19 mortality, social expectations about distance, and social expectations about sanctioning in Rome (Italy), June 2021–Feb 2022. Panel (*a*) plots the mortality trend as the logarithm of the mortality in the week before each wave’s survey was administered; panel (*b*) plots the average empirical and normative expectations about the distance people in Rome keep and think one should keep when in line at the supermarket; panel (*c*) plots the average empirical and normative expectations about the distance at which people in Rome would sanction or think that one should. The waves are plotted on the *x*-axis, corrected for the actual time that passed between each wave. In panels *b*,*c*, the triangles indicate the expected social expectations if the predicted trend from waves 1–5 were to continue the same way in waves 6–10. (Online version in colour.)

From June to August 2021 the COVID-19 mortality in Lazio (Italy) dropped from 74 deaths in a week to only 7. By mid-October 2021, death rate had increased again (25 deaths in one week) and it continued to increase to reach 123 deaths in one week by the first week of February 2022 (figure 2*a*). Throughout this entire period, official Italian government regulations specified to maintain at least 1 m distance from others in public places. This is reflected by the respondent’s normative expectations about the distance others think one ought to maintain in line at the supermarket. While there are slight fluctuations (the average normative expectations vary between 104 and 112 cm across all 10 waves), for the full study period they never drop below the prescribed 1 m (figure 2*b*). Empirical expectations of distance do drop below 1 m. They start at 103 cm and drop to 95 cm by August. In the second study period, as risk increases, initially empirical expectations continue to decrease, reaching their lowest point (92 cm) by mid-November 2021 (wave 7). Afterwards, there is a slight increase, but they never recover to levels on or above 1 m. The steepest changes occur for empirical and normative expectations of sanctioning (which follow the same pattern). In general, over the full study period, people expect others to tolerate distances about 10–15 cm smaller than the average expected distance maintained before intervening. Both empirical and normative expectations of sanctioning start at 91 cm in June 2021 and drop to ≈80 cm by August. They increase from ≈80 cm to 85 cm between October and January, only to drop to 80 cm again by the beginning of February 2022 (figure 2*c*). All in all, if we qualitatively observe the trends it seems that while there are some fluctuations in social expectations that follow changes in risk, expectations are late to recover when risk increases.

Multilevel OLS regressions confirm this observation (see table 2 for a summary of the main effects and tables S3–S6 in the electronic supplementary material for detailed results including the effects of the control variables). In line with Hypothesis 1, in waves 1–5 (the period of risk decrease), there was a positive relationship between mortality and empirical expectations of distance (*b* = 2.873, *p* = 0.049), empirical expectations of sanctioning (*b* = 5.327, *p* = 0.001), and normative expectations of sanctioning (*b* = 4.653, *p* = 0.003), such that all three decrease when risk decreases. However, contrary to Hypothesis 1 there was no significant relationship between COVID-19 risk and normative expectations of distance (*b* = 0.759, *p* = 0.545).

**Table 2.** Multilevel OLS regression of COVID-19 risk on social expectations. Standard errors in parentheses. All models control for gender, age, educational degree, political preferences, perceived health, previous COVID-19 infection, risk preferences, generalized trust, trust in politicians, trust in doctors and trust in scientists. See electronic supplementary material, tables S3–S6 for detailed results including control variables.

	M1: waves 1–5	M2a: all	M2b: all
	(*N* = 749)	(*N* = 1700)	(*N* = 1700)
*DV: empirical expectations of distance*			
intercept	90.472^***^	97.292^***^	92.307^***^
	(4.764)	(4.466)	(3.659)
log(new deaths *t* − 1)	2.873*	0.140	2.601*
	(1.461)	(1.206)	(1.123)
waves 6–10			−7.207^***^
			(1.796)
*DV: normative expectations of distance*			
intercept	107.215^***^	111.432^***^	107.595^***^
	(4.091)	(4.321)	(3.187)
log(new deaths *t* − 1)	0.759	−0.914	0.978
	(1.254)	(1.168)	(0.978)
waves 6–10			−5.430^***^
			(1.565)
*DV: empirical expectations of sanctioning*			
intercept	70.459^***^	76.842^***^	73.743^***^
	(5.127)	(4.434)	(4.041)
log(new deaths *t* − 1)	5.327^***^	2.421*	3.958^**^
	(1.572)	(1.196)	(1.240)
waves 6–10			−4.501*
			(1.982)
*DV: normative expectations of sanctioning*			
intercept	74.594^***^	78.480^***^	73.914^***^
	(5.106)	(4.671)	(3.925)
log(new deaths *t* − 1)	4.653^**^	1.938	4.199^***^
	(1.565)	(1.261)	(1.205)
waves 6–10			−6.634^***^
			(1.927)

*p < 0.05, **p < 0.01, ***p < 0.001.

Extrapolating on these results, we would expect empirical expectations of distance to be around 98 cm in wave 6, normative expectations around 111 cm, and empirical and normative expectations of sanctioning around 85 cm. From wave 6 to wave 10, we would expect normative expectations of distance to remain more or less constant, while all other expectations should slowly increase (see the dashed lines in figure 2*b*,*c*). Instead, when we estimate the effect of COVID-19 mortality on each of the social expectations for the full study period, COVID-19 risk only explains variation in the empirical expectations of sanctioning (*b* = 2.421, *p* = 0.043), although the relation is substantially weaker than for the period of risk decrease alone (a 2.5 cm change compared to a 5.3 cm change in empirical expectations for each log change in the number of deaths). All other social expectations do not significantly vary with the COVID-19 mortality (empirical expectations of distance: *b* = 0.140, *p* = 0.908; normative expectations of distance: *b* = −0.914, *p* = 0.434; normative expectations of sanctioning: *b* = 1.938, *p* = 0.124). This would be more in line with the hypothesis of norm erosion (H2b) than that of norms being adaptive (H2a).

However, if we compare the predicted trend of the social expectations to the observed trend (i.e. the dashed line to the solid line in figure 2*b* and *c*)*,* it seems that the rate of change is largely similar, but that the observed social expectations start at a lower level. Indeed, if we correct the intercept by controlling for the data collection period (table 2, M2b), we see that social expectations of all indicators are significantly lower in waves 6–10 (period of risk increase) than in waves 1–5 (empirical expectations of distance: *b* = −7.207, *p* < 0.001; normative expectations of distance: *b* = −5.430, *p* = 0.001; empirical expectations of sanctioning: *b* = −4.501, *p* = 0.023; normative expectations of sanctioning: *b* = −6.634, *p* = 0.001). Correcting for this lower starting point, we find positive relations between COVID-19 mortality and empirical expectations of distance (*b* = 2.601, *p* = 0.021), empirical expectations of sanctioning (*b* = 3.958, *p* = 0.001), and normative expectations of sanctioning (*b* = 4.200, *p* < 0.001). These expectations decrease when risk decreases and increase when risk increases, although they start increasing with some delay. While COVID-19 risk started rising between wave 5 and 6 already, social expectations started recovering only from wave 6 or 7 onward (and never did completely).

This evidence does not provide support for Hypothesis 2b that social norms erode after a decline in risk and are unable to recover once the risk rises again; but these findings do not provide full support for Hypothesis 2a either. Even though social expectations increase after the risk recurs, they are lower than the new risk level would require, making them less effective as solutions to the collective action problem. As a robustness check we repeated the same analyses using the perceived COVID-19 risk, rather than the mortality, as predictor variable. Perceived risk likely mediates the relationship between objective risk and respondents’ social expectations. For the full period, we found perceived risk to be positively associated with all four social expectations, which further strengthens our results (see electronic supplementary material, section S3.3 for detailed results).

### (b) From norms to behaviour

**Figure 3.**
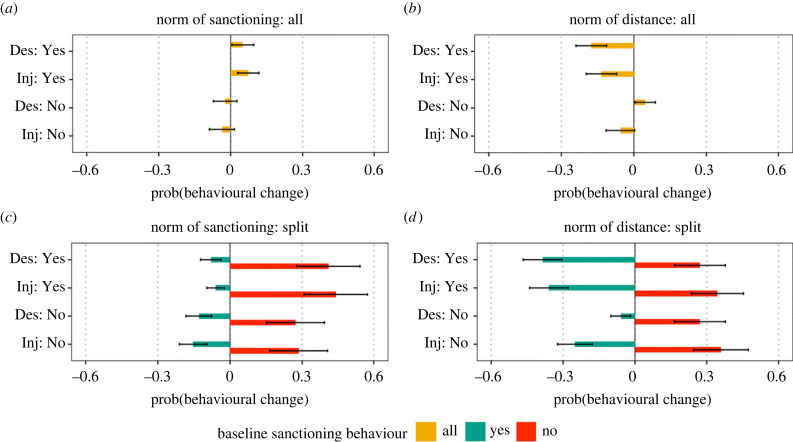
Effects of different descriptive and injunctive norms on intentions to sanction. Panel (*a*) plots the probability that sanctioning increases (or decreases) for the two components ([*descriptive/injunctive*]) of the meta norm of sanctioning in case a norm is present (Majority: Yes) or not (Majority: No). Panel (*b*) plots the same but with respect to the two components of the social norm of distance. Panels (*c*,*d*) plot for the same norms the probability that respondents who reported to sanction ('yes') and respondents who reported not to ('no') change their behaviour in the hypothetical scenario. (Online version in colour.)

Table 3 and figure 3 show how sanctioning intentions respond to different hypothetical social norms and meta norms. If the majority of people would sanction someone who keeps less than 1 m distance in line (i.e. a descriptive norm of sanctioning), the probability that someone is willing to sanction increases by *b* = 0.050 compared to the baseline reported sanctioning behaviour (*p* = 0.029). If the norm would be an injunctive norm (i.e. you know that a majority approves of sanctioning in such situations), the probability that someone sanctions increases by *b* = 0.073 (*p* = 0.001). While this is in line with Hypothesis 3a, the same does not hold for the absence of a meta norm of sanctioning. Neither knowing that a majority would not sanction (*b* = −0.023, *p* = 0.353) nor knowing that a majority would not approve of sanctioning (*b* = −0.037, *p* = 0.160) significantly reduced the probability that someone sanctions. A possible explanation is that the respondents’ reported empirical and normative expectations of sanctioning are in line with these hypothetical norms, so these hypothetical scenarios do not differ from their baseline behaviour.

**Table 3.** Probability of sanctioning conditional on type of social information and baseline sanctioning behaviour. Standard errors in parentheses. All models control for gender, age, educational degree, political preferences, perceived health, previous COVID-19 infection, risk preferences, generalized trust, trust in politicians, trust in doctors, and trust in scientists. See electronic supplementary material, tables S15–S18 for detailed results of underlying OLS regression models including control variables.

	sanctioning:		distance:	
	descriptive	injunctive	descriptive	injunctive
scenario: majority yes				
all	0.050*	0.073^**^	−0.176^***^	−0.134^***^
	(0.023)	(0.024)	(0.032)	(0.031)
baseline: sanctions	−0.080^**^	−0.060^*^	−0.386^***^	−0.359^***^
	(0.021)	(0.018)	(0.040)	(0.040)
baseline: does not sanction	0.410^***^	0.441^***^	0.272^***^	0.345^***^
	(0.066)	(0.066)	(0.053)	(0.055)
scenario: majority no				
all	−0.023	−0.037	0.046	−0.056
	(0.025)	(0.026)	(0.021)	(0.030)
baseline: sanctions	−0.130^***^	−0.154^***^	−0.060	−0.251^***^
	(0.027)	(0.029)	(0.021)	(0.036)
baseline: does not sanction	0.272^***^	0.286^***^	0.272^***^	0.360^***^
	(0.060)	(0.060)	(0.053)	(0.057)

*p < 0.05, **p < 0.01, ***p < 0.001.

Contrary to our expectations, sanctioning intentions do not increase when people are told that there is a social norm of distance in place. Instead, when they would know that the majority of others maintains at least 1 m distance, the probability that someone sanctions a violation of this rule decreases by *b* = −0.176 (*p* < 0.001). Being told that the majority of others would not keep distance would increase sanctioning intentions (*b* = 0.046, *p* = 0.031). When they would know that a majority of others thinks people should maintain at least 1 m distance, they are likewise less likely to sanction violations (*b* = −0.134, *p* < 0.001), although in this case the lack of an injunctive norm of distance does not significantly change sanctioning intentions (−0.056, *p* = 0.062). Altogether, these results reject Hypothesis 3b.

To explore why we find these contrasting results, we test an interaction with the baseline sanctioning behaviour. This reveals that different norm scenarios invoke different responses by different subgroups. People that reported not to sanction in general responded most strongly to the hypothetical scenario that others believe one should sanction violations of the 1 m distance rule (i.e. an injunctive meta norm): this makes someone who did not sanction before 44% more likely to do so (*b* = 0.441, *p* < 0.001). However, a specific social norm or meta norm may also decrease sanctioning behaviour for some of the people who initially reported to sanction violations. This probability is small for a descriptive (*b* = −0.060, *p* = 0.004) or injunctive (=−0.060, = 0.001) norm of sanctioning, which is why the positive change of people who start sanctioning outweighs the negative change of people who stop sanctioning. Knowing that a descriptive or injunctive norm of distance is in place, on the other hand, makes more sanctioning people inclined to stop sanctioning (descriptive: *b* = −0.386, *p* < 0.001; injunctive: *b* = −0.359, *p* < 0.001) than it convinces non-sanctioning people to start doing so (descriptive: *b* = 0.272, *p* < 0.001; injunctive: *b* = 0.345, *p* < 0.001). Overall, this results in a net negative effect.

The motivation given by respondents who did not sanction before but indicate that they would if a meta norm of sanctioning or a social norm of distance is in place is often in line with the theory. Those who become willing to sanction if a meta norm of sanctioning were in place say that such a norm would reassure them they are not alone in intervening, that it means others would support their intervention, or that they would not have to fear violent reactions. One respondent said, for instance: ‘I often have difficulty asserting my rights and perhaps, if in some way supported by others, I might be able to do so.’ Respondents who change their sanctioning behaviour based on the knowledge that a social norm of distance would be in place motivate this choice by saying it is easier to intervene if they know it is the socially desirable behaviour. To illustrate, one person said that by knowing the majority of others keeps distance ‘I would feel more secure in making my reasons heard’. This would imply that sanctioning behaviour indeed follows similar norm dynamics (both in response to a meta norm and in response to a social norm). Interestingly, while sanctioning is an individual act, it seems that respondents interpret it as a collective effort: they do so if they know others will join them or support their intervention if needed.

On the other hand, people who stop sanctioning under the hypothetical scenario that a meta norm of sanctioning is in place might rather see sanctioning behaviour as a volunteer’s dilemma: they motivate their behavioural change by saying they prefer not to sanction, but do so only if others do not. Knowing that a sanctioning norm is in place makes them believe someone else will intervene. They fear negative retaliation by the norm violator, so if they can avoid sanctioning themselves they would. Or, in the words of one of the respondents: ‘Without the support of others intervening could become dangerous’. If a social norm of distance is in place, several respondents indicate that in such a situation it is not necessary to intervene, that sometimes one should accept smaller distances, or that we should have some tolerance. In other words, if a social norm of distance is in place, the observed violation would be an exception, so those respondents who indicate they would stop sanctioning believe it is not necessary to strengthen the norm through social sanctions.

In general, fear for retaliation was a recurring theme for respondents to change their intervention intentions, regardless of the hypothetical norm that we presented. Many respondents indicated that the COVID-19 health crisis made people more nervous, that those who do not comply with preventative measures are usually also the ones that would respond aggressively, and that this makes them afraid to reprimand someone who violates the distance norm. One respondent said: ‘Honestly, I’m afraid of the reactions of the recipients of a potential reminder of the rules. [...] It’s certainly not something I’m proud of, but I’m afraid of making the situation worse, that’s my sincere feeling.’ A detailed list of respondents’ motivations is available in the electronic supplementary material.

## 5. Discussion

A hindrance to empirically studying norm change is that norm change typically occurs slowly. Observational studies thus often rely on cross-country comparisons, assuming that any difference in social norms between these countries, or regions within a country, are the result of a different history of norm evolution and change [23,26,27]. An experimental setting offers more control to study the relation between a changing context (such as risk) and the effect on norms [32], yet creates an artificial, less realistic setting that requires norms to be created and then change in response to some experimental manipulation within a limited period of time. The COVID-19 pandemic, which required fast, large-scale behavioural change and the establishment of new social norms, offered a unique opportunity to study processes of norm formation and decay in a real-world setting due to its evolution characterized by multiple changes in risk.

We measured empirical and normative expectations about social distancing in public spaces and empirical and normative expectations about sanctioning distance violations from June 2021 to February 2022 to see how norms and meta norms evolved as the COVID-19 risk decreased and increased. Our objectives were to study the dynamics of social norms and meta norms over recurrent changes in risk, to understand how resilient norms and meta norms are to changes in risk, and to test whether norm nudges can increase peer sanctioning of norm violations.

We found some support for the conjecture behind the tightness–looseness theory [23] of the link [between the severity of threats faced by a society and the strength of its social norms. Empirical expectations of distance and empirical and social expectations of sanctioning first decreased with COVID-19 risk and subsequently increased again, suggesting that tightening and loosening of social norms is not only a long-term evolutionary process, but can be observed at shorter timescales as well. Importantly, our results indicate that the loosening of social norms does not initiate a process of norm erosion and abandonment. In situations of recurrent risks—like the one posed by the COVID-19 pandemic—social norms regulating social distance and meta norms regulating the responses to [their violation are (to some extent) able to tighten up again when the situation requires it.

However, while this suggests that social norms are adaptive, we also observed that this collective responsiveness occurs with a dangerous delay. COVID-19 risk had been on the rise for a couple of weeks already before social norms started adapting. Hence, while norms are capable of spontaneously adapting to exogenous circumstances, this process is far from perfect. Social norms and meta norms may easily lose force when risk decreases and compliance is less advantageous, but struggle more to strengthen again when the situation requires it. In other words, while norms do not erode there does seem to be an asymmetry in that the weakening of norms goes faster than their strengthening (see also [24]). For changes in risk within short time frames, social norms are therefore only partially effective in guiding cooperative behaviour, as they may not recover in time in the face of new threats. This inertia to change may call for external interventions to speed up the process.

A reasonable way to facilitate the tightening process is to reduce the tolerance for norm violations that the decrease in risk had made more widespread. Since we adopted the view that sanctioning is shaped by its own social expectations and observed a decline in meta norms when the risk decreases, we also tested several hypothetical norm nudges to see how they might reduce tolerance for the violation of social distancing norms. An increased tolerance for norm violations may in fact hinder a fast norm recovery when risk recurs. When people expect others not to sanction norm violations or believe that one should, they might be less likely to comply with the social norm despite the increased risk—and expect others to do the same. Norm nudges have been widely adopted to increase compliance with social norms in the context of collective action [49], although the conditions under which they are effective is still disputed.

Supporting our expectations and consistent with the large literature on norm nudges, providing respondents with information about the existence of a hypothetical strong meta norm of sanctioning (with descriptive and injunctive content) increased sanctioning intentions. Importantly, however, the same does not hold for norm nudges strengthening the social norm of distance itself. Informing respondents that the majority of people keeps at least 1 m distance from others or believes that one should reduced the respondents’ willingness to sanction a violation of this norm. Further analyses indicated that while some of the (previously not-sanctioning) respondents do change their sanctioning intentions in this setting, the net negative effect is driven by a backfiring effect on those respondents that already sanctioned. Knowing that a strong social norm of sanctioning is in place strongly reduced their willingness to sanction. While our norm nudges reflect hypothetical scenarios and unincentivized behavioural intentions, they do warrant caution in the use of norm nudges. Informing people that norm compliance is higher than they think it is, likewise means informing them that the number of violations is smaller than they expect it to be. This, in turn, might make them feel that there is less need to enforce the norm, which could in the long-term lead to norm decay.

Altogether, these real-world dynamics provide important insights into the (in)stability of social norms, about when norms are useful to deal with social dilemmas under risk, and when instead they are not. Our visual measure of distance between others allowed for a more reliable elicitation of social expectations than a more general or abstractly worded question [48] and thus made it possible to track changes in expectations over time on a continuous scale. Moreover, we contribute to the existing literature on norm nudging by investigating how to sanction intentions change following norm information.

There are, however, some limitations to be kept in mind. First, while we have studied how expectations about the social norm of distance and meta norm of sanctioning change with risk, we do not know how these fluctuations have influenced actual behaviour. The changes in empirical expectations give some indication, but we do not know how social expectations of the existence or lack of a social or meta norm (causally) influenced the behaviour of people. Second, while we tested for the relation between social norms, meta norms and sanctioning intentions, these tests reflect hypothetical scenarios and might not reflect real-life behavioural change. Third, we cannot exclude that changes in perceived norms and meta norms that we attributed to variations in COVID-19 risk do not also depend on other, unobserved factors that may bias our results. For example, the two periods of time in which we collected the data correspond to seasons with different characteristics, namely summer and winter. Seasonal variation and climate-related aspects might have influenced changes in risk perception and norms. Recent studies [50,51] show that seasons might affect psychological phenomena, including mood, aggressiveness and prosocial behaviour. Moreover, seasonal variation also impacts the incidence of infectious diseases [52], and this has been reported to have an effect on the activation of the motivational system that regulates behavioural defence against infection, the ‘behavioural immune system’ [53]. This activation might have consequences for risk perceptions. For example, people might perceive the risks they face as more severe and become more pessimistic about how others behave to cope with these risks.

Nonetheless, at least qualitatively, the trends observed in this study stress the importance of considering not only how social norms change behaviour, but also how social norms themselves change over time or in response to changing contexts. Future research should take an integrated approach to the study of risk, norms and behaviour by acknowledging the dynamic feedback loops that characterize their underlying relationship. Moreover, with respect to norm nudges, our findings highlight the importance of evaluating nudges’ distributional effects [54] that requires a diagnosis of the social environment and social norms underlying the behaviour that they aim to change.

## Data Availability

The data and scripts to reproduce the results are publicly available from the OSF repository OSF: https://osf.io/6fh4v [55]. Electronic supplementary material is available online [56].
